# Supplementary figures and images for: Protein Phosphatase 1 Down Regulates ZYG-1 Levels to Limit Centriole Duplication
Source: PLoS Genet. 2017 Jan 19;13(1):e1006543. doi: 10.1371/journal.pgen.1006543 (PMC5289615; doi:10.1371/journal.pgen.1006543)

Supplemental Figure S1

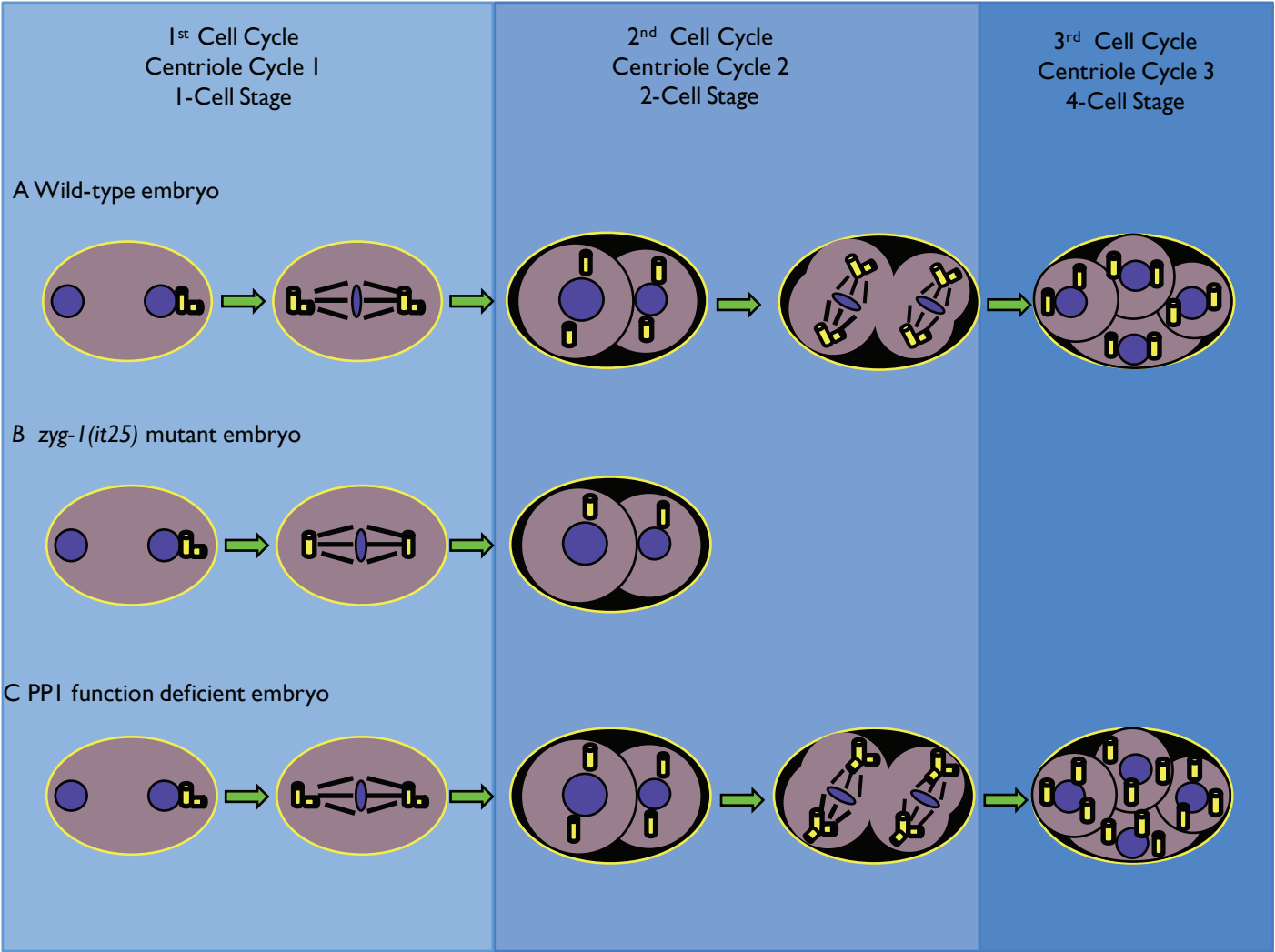

Supplement: S1 Fig — Schematic showing how centrioles behave and how their numbers are established at each cell stage through the first three cell cycles in A) wild-type embryos, B) zyg-1(it25) mutant embryos and in C) embryos with reduced PP1 function. (PDF) [file pgen.1006543.s001.pdf]

Supplemental Figure S2

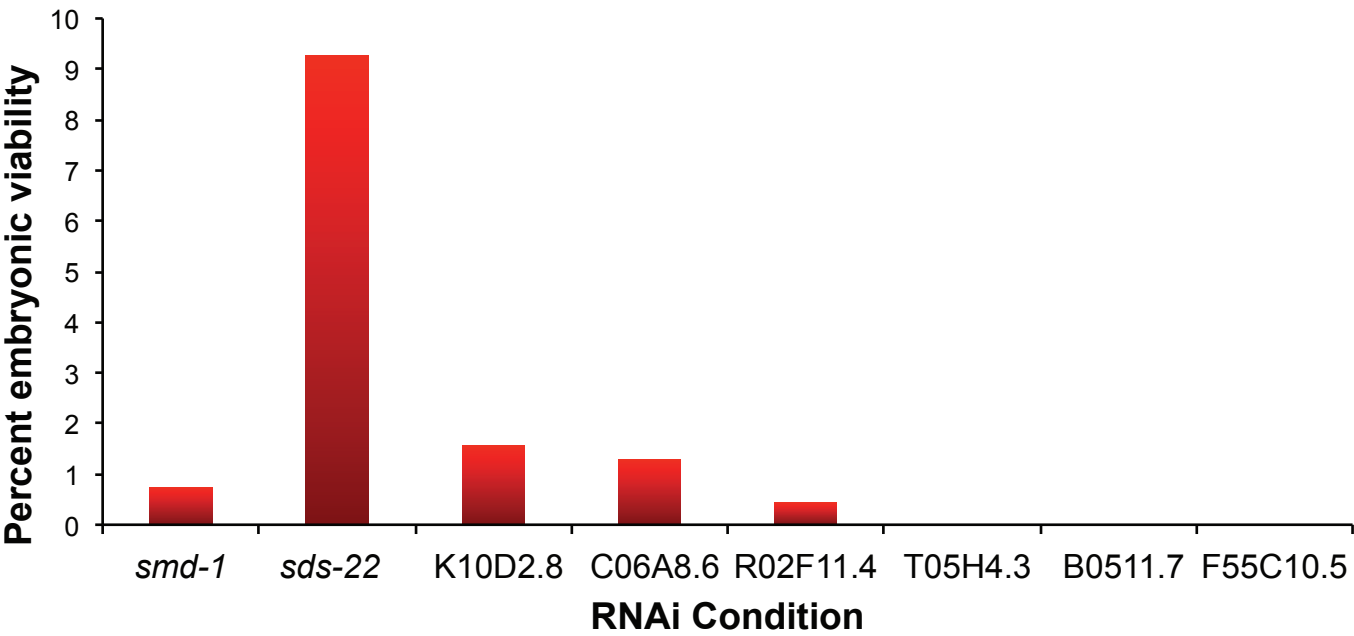

Supplement: S2 Fig — Quantification of embryonic viability among the progeny of worms grown at the semi-permissive temperature of 24°C and depleted for the indicated PP1 regulators. smd-1(RNAi) targets a nonessential gene and serves as a negative control. Only reduction of sds-22 led to substantial rescue of zyg-1(it25) lethality. (PDF) [file pgen.1006543.s002.pdf]

Supplemental Figure S3

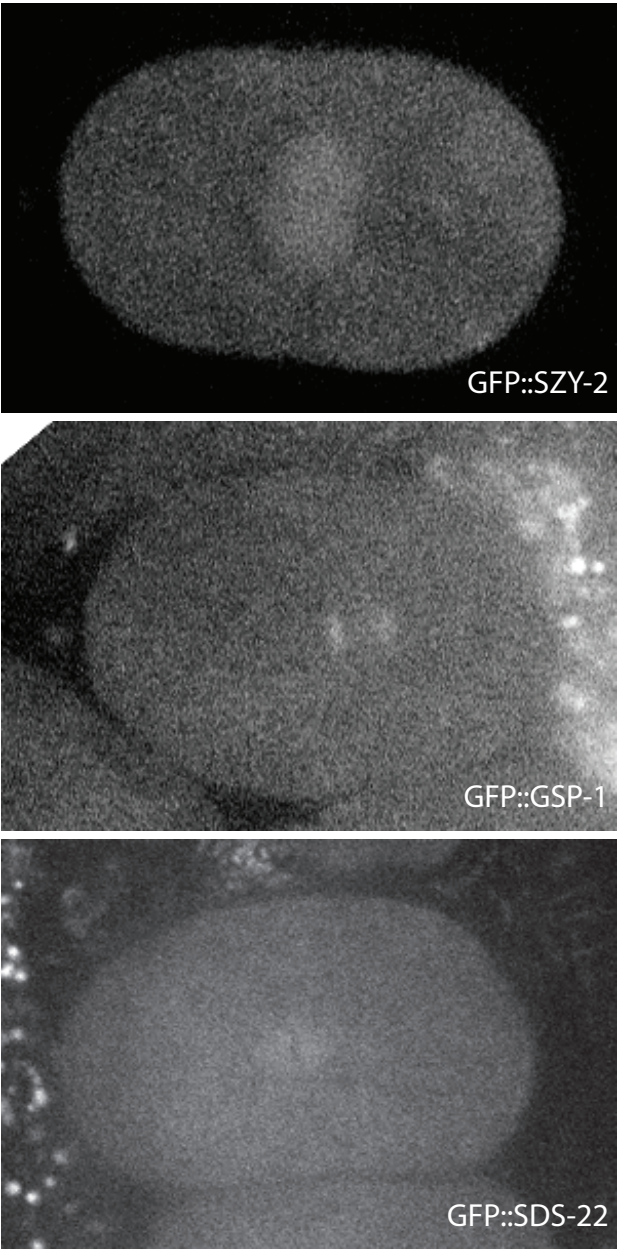

Supplement: S3 Fig — GFP fusions of the indicated protein were expressed in the embryo. I-2SZY-2 is enriched in the nuclei. Pronuclei meeting during the first cell cycle is shown. PP1βGSP-1 is enriched on the chromatin throughout mitosis. Anaphase of the first cell cycle is shown. SDS-22 is weakly localized to the spindle. Metaphase of the first cell cycle is shown. (PDF) [file pgen.1006543.s003.pdf]

Supplemental Figure S5

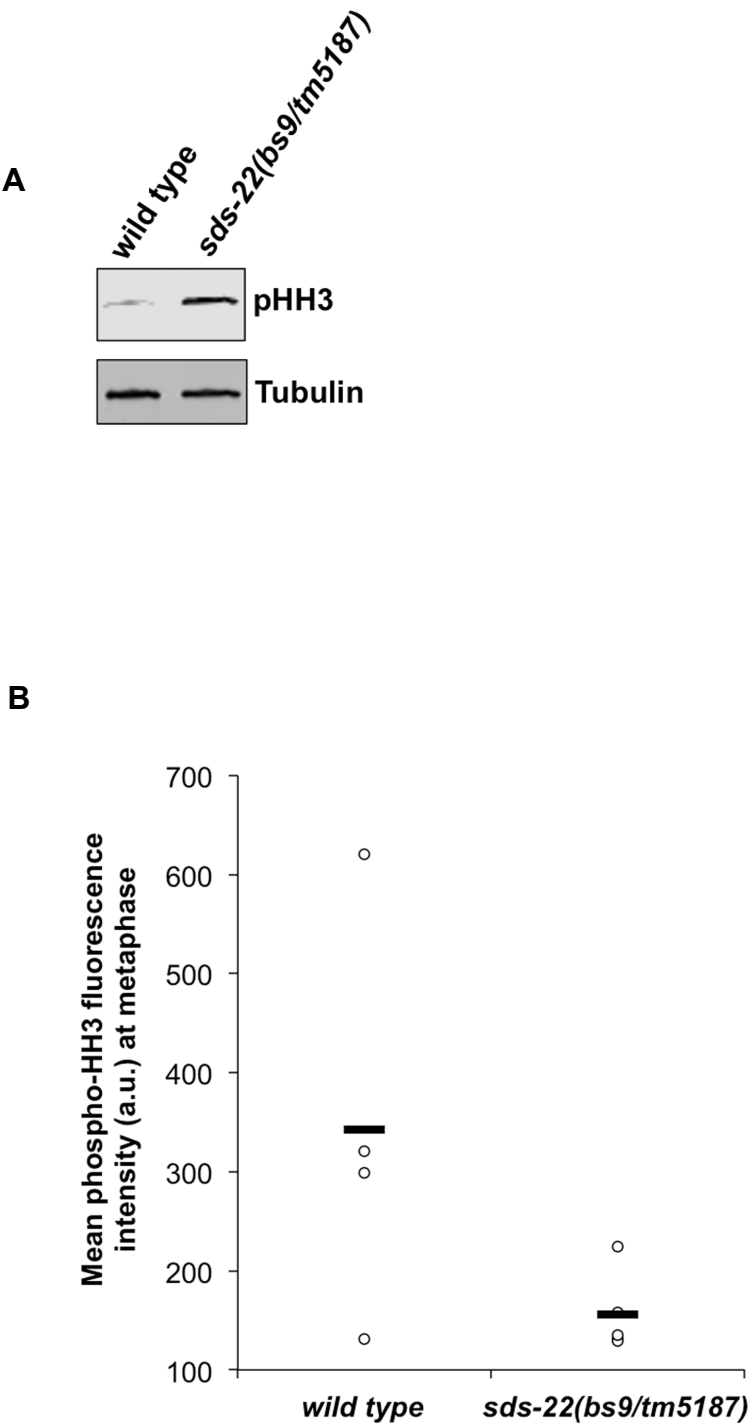

Supplement: S5 Fig — A) Western blot showing elevated total phospho-histone levels present in extract from mixed stage sds-22(b9/tm5187) embryos. This elevation is likely due to an increase in the length of mitosis in sds-22 mutant embryos as B) quantitative immunofluorescence microscopy shows that mitotic chromatin in sds-22(bs9/tm5187) embryos is not enriched for phospho-histone H3 relative to the wild type, and in fact, appears reduced. (a.u. = arbitrary units). (PDF) [file pgen.1006543.s005.pdf]

Supplementary Figure S6

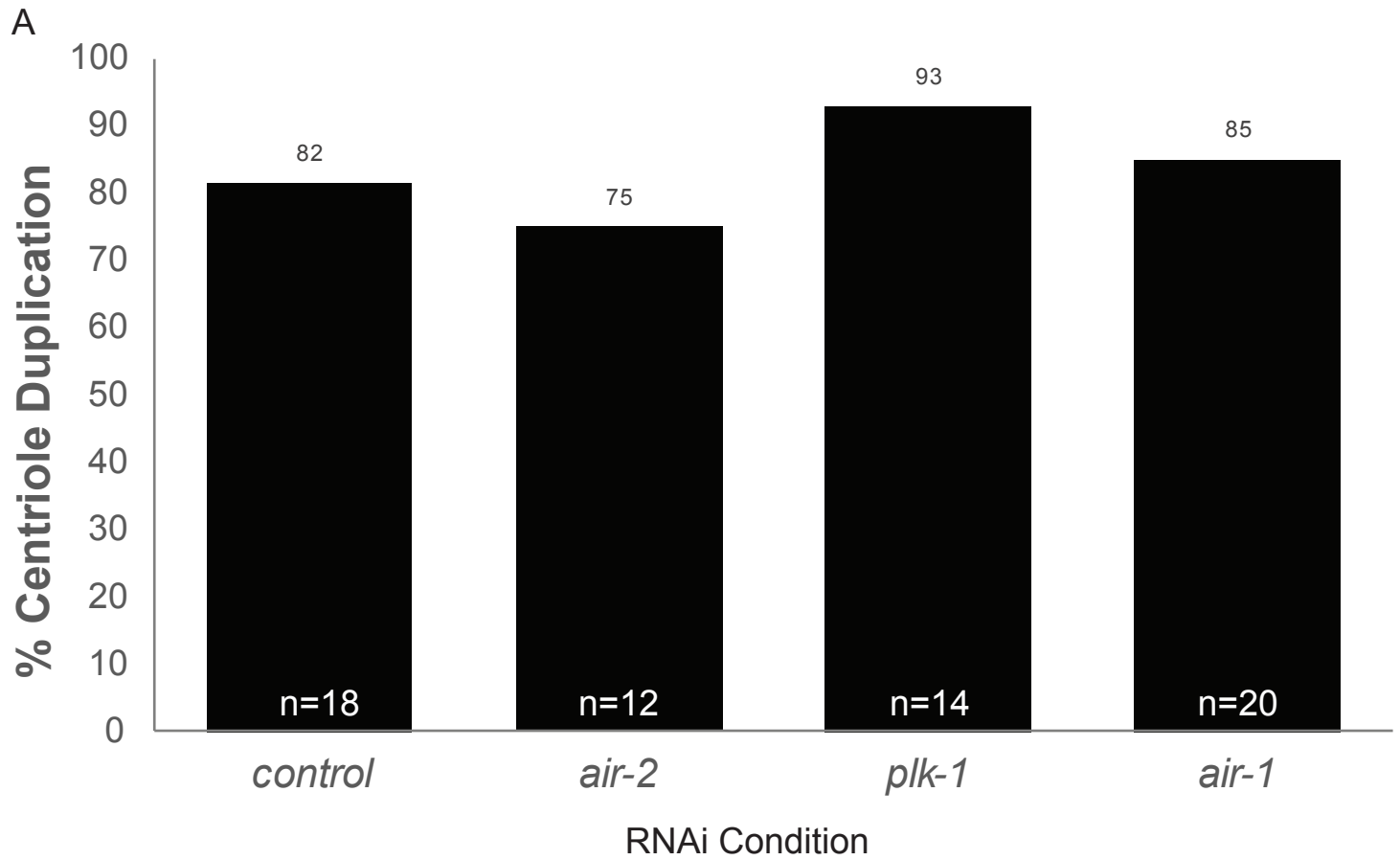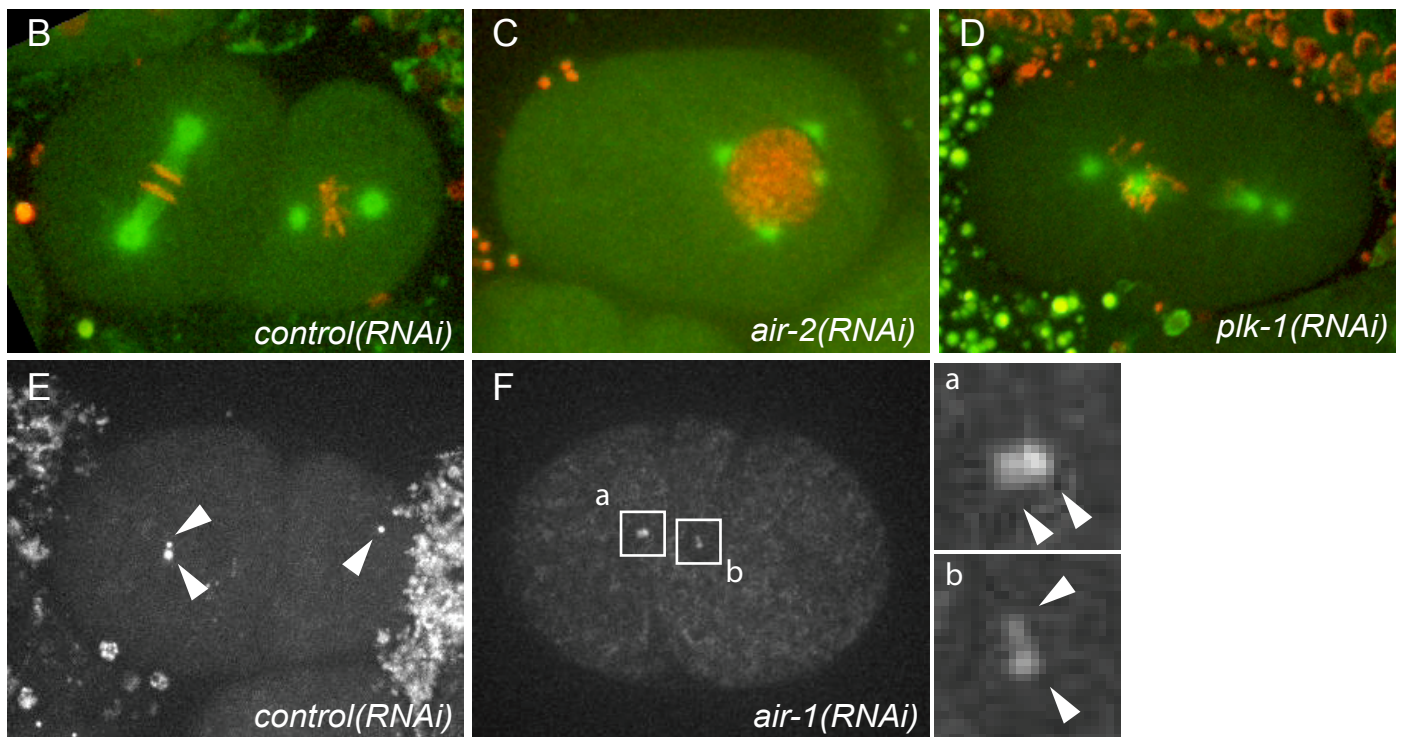

Supplement: S6 Fig — Each of the specified kinases were RNAi depleted in zyg-1(i25); szy-2(bs4) worms and centriole duplication monitored by microscopy. A) Quantification of centriole duplication. Numbers above bars indicate the percentage of successful centriole duplication events and the number within the bars indicate the number of events scored. B-D) Representative stills from time-lapse recordings of zyg-1(it25); szy-2(bs4) embryos expressing GFP::tubulin and mCherry::histone and treated with control RNAi or RNAi against one of the three indicated mitotic kinases. B) Embryos treated with control RNAi duplicate centrioles, proceed to the 2 cell stage, and build bipolar spindles. C) Embryos treated with air-2 RNAi duplicate centrioles, but fail in chromosome segregation, and thus display 4 centrosomes associated with a single enlarged nucleus. D) Embryos treated with plk-1 RNAi show a variety of cell division defects yet present with 4 centrosomes indicating centriole duplication has occurred. E&F) Representative stills from time-lapse recordings of zyg-1(it25); szy-2(bs4) embryos expressing GFP::SPD-2 and treated with control RNAi (E) or RNAi against air-1 (F). Centrioles from F are enlarged in a&b. Embryos are at the early 2-cell stage and duplicated centrioles are visible (arrow heads). Note, in this example only one centriole of the control embryo has duplicated. (PDF) [file pgen.1006543.s006.pdf]

Supplemental Figure S7

A

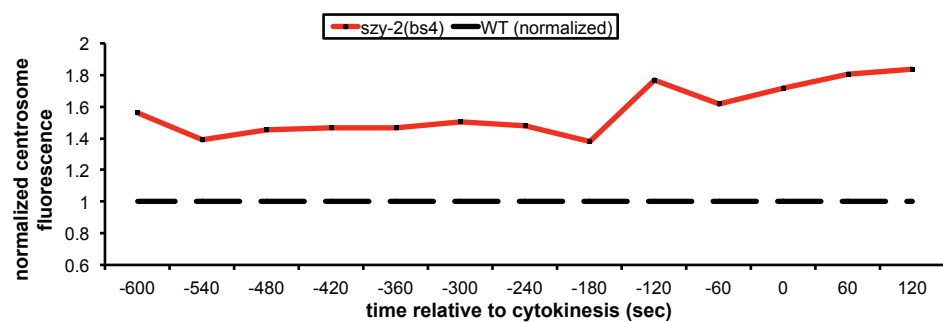

B

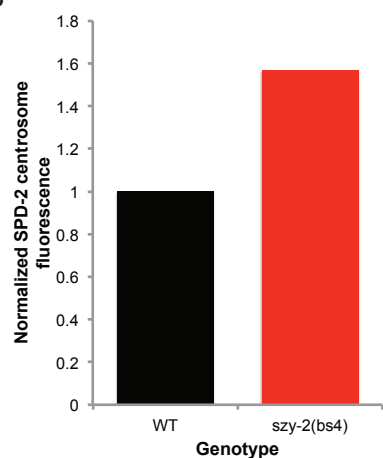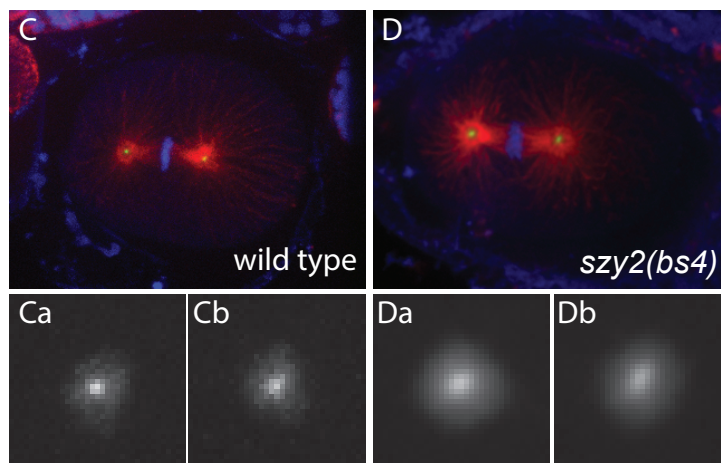

G

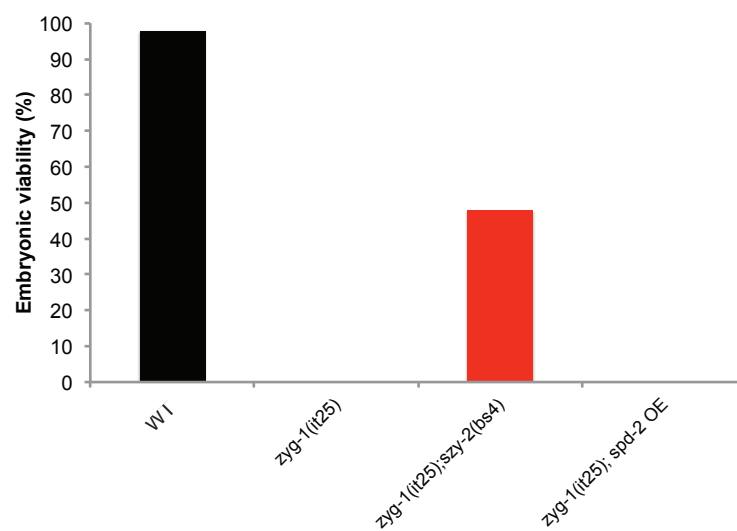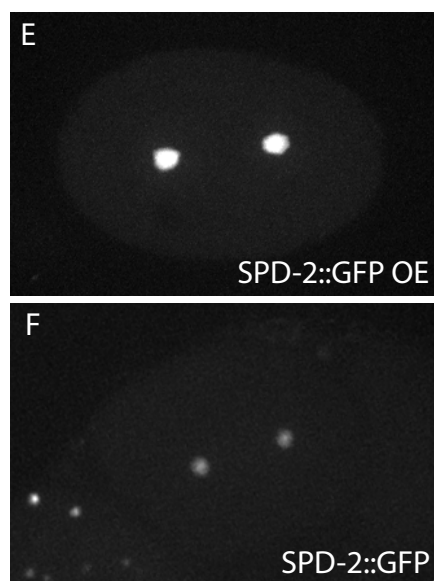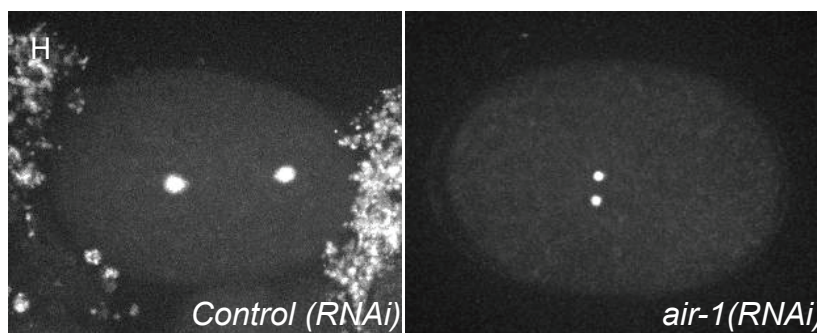

Supplement: S7 Fig — A) Average GFP::SPD-2 levels at the centrosome during the first cell cycle were calculated and normalized to control. B) Average levels of endogenous SPD-2 at the centrosome during the first metaphase in szy-2(bs4) embryos were normalized to controls. C&D) Representative wild-type and szy-2(bs4) embryos stained for DNA (blue), tubulin (red) and SPD-2 (green). SPD-2 staining at centrosomes is enlarged in a and b. E&F) Comparison of SPD-2::GFP levels at the centrosome in an embryo expressing a codon-optimized version of SPD-2::GFP (OE = overexpression) (E) and a strain expressing GFP::SPD-2 from the native sequence (F). G) Quantification of embryonic viability among the indicated strains to determine whether overexpression of SPD-2 is sufficient to rescue the zyg-1(it25) phenotype. In each case n = 15 worms, >1000 embryos. H) GFP::SPD-2 levels at the centrosome in zyg-1(it25); szy-2(bs4) embryos treated with negative control smd-1(RNAi) or air-1(RNAi). (PDF) [file pgen.1006543.s007.pdf]

Supplemental Figure S8

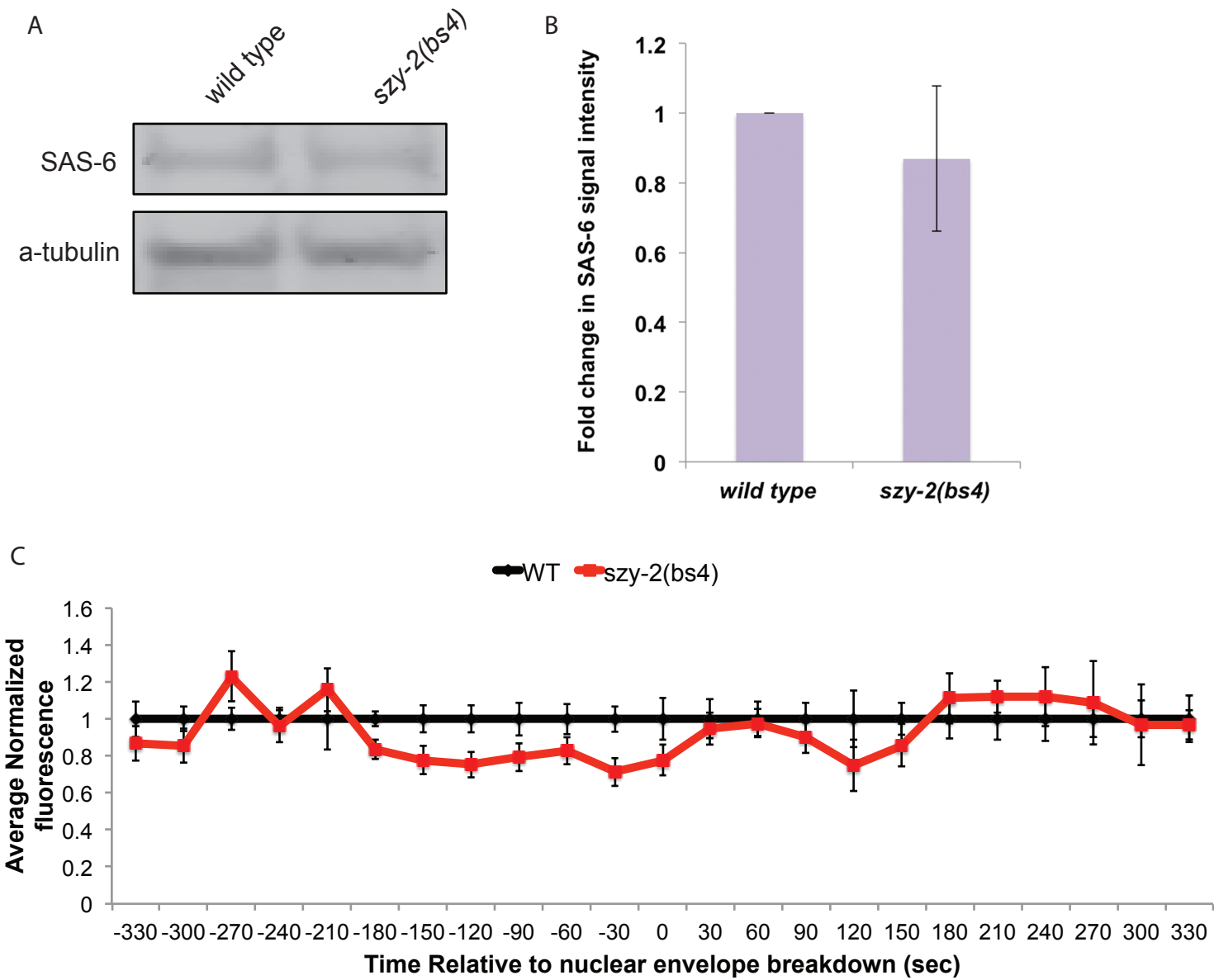

Supplement: S8 Fig — A) Western blot demonstrating equivalent total levels of SAS-6 in wild-type and szy-2(bs4) embryos. B) Quantitation of western blot data (n = 2). C) Average levels of centrosome-localized GFP::SAS-6 in wild-type and szy-2(bs4) embryos were determined throughout the first cell cycle. Values shown are normalized to the average wild-type value at each stage. Error bars represent standard error. (PDF) [file pgen.1006543.s008.pdf]

Supplemental Figure S9

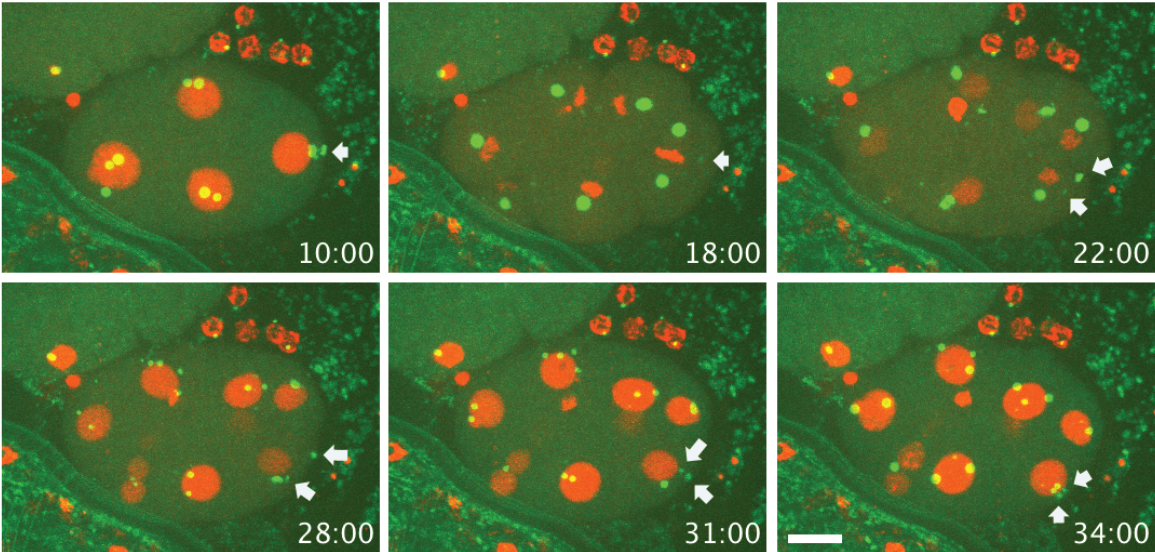

Supplement: S9 Fig — Select frames from a time-lapse recording of an sds-22(bs9/tm5187) embryo expressing GFP::SPD-2 and mCherry::histone. The arrowhead shows a spindle pole (frame 18:00) giving rise to multiple centrosomes during the next cell cycle (frame 28:00). White arrows indicate centrosomes that become inactive during the round of division following their birth (frames 10:00 and 18:00) but become active again during the next cell cycle (frames 22:00–34:00). Note that both centrosomes were not always visible in these frames. (PDF) [file pgen.1006543.s009.pdf]
