# Supplementary material for: Protein Phosphatase 1 Down Regulates ZYG-1 Levels to Limit Centriole Duplication
Source: PLoS Genet. 2017 Jan 19;13(1):e1006543. doi: 10.1371/journal.pgen.1006543 (PMC5289615; doi:10.1371/journal.pgen.1006543)
Supplement: S4 Fig — Each of the indicated proteins was immunoprecipitated from worm extracts and co-purifying proteins identified by mass spec. Shown are the top five hits based on peptide number. In cases where PP1GSP-1, SDS-22 or I-2SZY-2 were not among the top hits, they are also shown along with their rank and number of identifying peptides. (PDF) [file pgen.1006543.s004.pdf]

Supplemental Figure S4

PP1β^{GSP-1} IP mass spec

Gene Name	Number of peptides	Rank
nmy-2	67	1st
pab-1	31	2nd
atx-2	26	3rd
ani-1	24	4th
tcc-1	21	5th
sds-22	18	10th
gsp-1	16	14th
szy-2 (I-2)	10	50th

I-2^{SZY-2} IP mass spec

Gene Name	Number of peptides	Rank
gsp-2	17	1st
tbb-2	11	2nd
gsp-1	9	3rd
hsp-1	7	4th
pab-1	7	5th
szy-2 (I-2)	6	8th

SDS-22 IP mass spec

Gene Name	Number of peptides	Rank
tsn-1	13	1st
tbb-2	12	2nd
hsp-60	9	3rd
C08H9.2	9	4th
tcp-1	8	5th
sds-22	8	7th
gsp-2	6	16th
gsp-1	1	91st
