# Supplementary material for: Protein Phosphatase 1 Down Regulates ZYG-1 Levels to Limit Centriole Duplication
Source: PLoS Genet. 2017 Jan 19;13(1):e1006543. doi: 10.1371/journal.pgen.1006543 (PMC5289615; doi:10.1371/journal.pgen.1006543)
Supplement: S1 Table — (DOCX) [file pgen.1006543.s010.docx]

**Supplemental Table 1 *C. elegans* strains used in this study**

| Strain name | Genotype |
| --- | --- |
| OC467 | *szy-2(tm3972)/hT2[bli-4(e937) let?(q782) qIs48](I;III)* |
| OC192 | *szy-2(bs4) III* |
| OC227 | *zyg-1(it25)* II*; szy-2(bs4)* *III* |
| OC14 | *zyg-1(it25)* *II* |
| OC192 | *szy-2(bs4) III* |
| OC199 | *zyg-1(it25) sds-22(bs9) II* |
| OC367 | *zyg-1(it25) II; szy-2(bs4)* *unc-119(ed3) III*; *ltIs37[pAA64: unc-119(+) pie-1p::mcherry::his58]; ltIs25[pAZ132: unc-119(+) pie-1p::gfp::tba-2]* |
| OC429 | *dpy-17(e164)* *szy-2(bs4) III; bsIs2 [pCK5.5: : unc-119(+) pie-1p::gfp::spd-2]* |
| OC448 | *zyg-1(it25*) *II; ltIs37[pAA64: unc-119(+) pie-1p::mcherry::his-58]; ltIs25[pAZ132: unc-119(+) pie-1p::gfp::tba-2]* |
| OC469 | *zyg-1(it25) II; szy-2(tm3972) III; ltIs37[pAA64: unc-119(+) pie-1p::mcherry::his-58]; ltIs25[pAZ132: unc-119(+) pie-1p::gfp::tba-2]* |
| OC573 | *sds-22(tm5187)/mIn1[dpy-10(e128) mIs14(myo-2::gfp)] II* |
| OC156 | *zyg-1(it25) II;* *bsIs2 [pCK5.5: : unc-119(+) pie-1p::gfp::spd-2]; unc-119(ed3) ruIs32[unc-119(+) pie-1p::gfp::his-58] III* |
| OC381 | *zyg-1(it25) II; gsp-2(tm301)/ hT2[bli-4(e937) let?(q782) qIs48](I;III);* *ltIs37[pAA64: unc-119(+) pie-1p::mcherry::his-58]; ltIs25[pAZ132: unc-119(+) pie-1p::gfp::tba-2]* |
| OC626 | *sds-22(bs9)/sds-22(tm5187) II; bsIs2* *[pCK5.5: unc-119(+) pie-1p::gfp::spd-2]; ltIs37 [pAA64: unc-119(+) pie-1p::mcherry::his-58]* |
| OC591 | *zyg-1(it25);* [unc-119(+) *pie-1-GFP::SPD-2OE*]*Insertion from Decker et al, 2011* |
| OC92 | *unc-119(ed4) III; bsIs2 [pCK5.5: unc-119(+) pie-1p::gfp::spd-2]* |
| NIN33 | *szy-2(bs4) unc119(ed3) III; fem-1(hc17) IV; ItIs33[pOD224: unc-119(+) pie-1p::gfp::tev::stag::sas-6;]* |
| OC770 | *bsSi15 [pKO109: spd-2p::spd-2::mCherry::spd-2 3'-utr, unc-119(+)] I; szy-2(tm3972) III* |
| OC779 | *bsSi15 [pKO109: spd-2p::spd-2::mCherry::spd-2 3'-utr, unc-119(+)] I; bsSi30[pCW9: ZC504.3p::sfgfp::his-58::ZC504.3 3’-utr, unc-119(+)] II* |
| OC723 | *bsSi9 [pKO113: unc-119(+) zyg-1p::gfp::his-58::3'utr zyg-1] I; unc-119(ed3) III* |
| OC835 | *bsIs9 [pKO113: unc-119(+) zyg-1p-gfp::his-58-zyg-1 3'utr]I; szy-2(tm3972) III* |
| OC547 | *unc-119(ed3)III: bsIs33 [pNP121: pie-1promoter-gfp-sds-22-pie-1UTR unc-119(+)]/+* |
| OC484 | *unc-119(ed3) III; BsIs18[pNP97: pie1promoter-gfp-gsp-1genomic-pie1UTR unc-119(+)]* |
| OC426 | *unc119(ed3)III; pNP71: bsIs52[pie-1promoter-gfp::szy-2-pie-1UTR unc119(+)]* |
